# Placental growth factor, pregnancy-associated plasma protein-A, soluble receptor for advanced glycation end products, extracellular newly identified receptor for receptor for advanced glycation end products binding protein and high mobility group box 1 levels in patients with acute kidney injury: a cross sectional study

**DOI:** 10.1186/1471-2369-14-245

**Published:** 2013-11-04

**Authors:** Oskar Zakiyanov, Vitezslav Kriha, Jan Vachek, Tomas Zima, Vladimir Tesar, Marta Kalousova

**Affiliations:** 1 Department of Nephrology, First Faculty of Medicine, Charles University in Prague and General University Hospital in Prague, Prague, Czech Republic; 2 Institute of Medical Biochemistry and Laboratory Diagnostics, First Faculty of Medicine, Charles University in Prague and General University Hospital in Prague, Prague, Czech Republic; 3 Department of Physics, Faculty of Electrical Engineering, Czech Technical University in Prague, Prague, Czech Republic; 4 Institute of Pharmacology, First Faculty of Medicine, Charles University in Prague, Prague, Czech Republic

**Keywords:** Acute kidney injury, Biomarkers, Chronic kidney disease, Extracellular newly identified receptor for advanced glycation end products binding protein, Haemodialysis, Placental growth factor, Pregnancy-associated plasma protein-A, Soluble receptor for advanced glycation end products

## Abstract

**Background:**

Placental growth factor (PlGF), pregnancy-associated plasma protein-A (PAPP-A), soluble receptor for advanced glycation end products (sRAGE), extracellular newly identified receptor for RAGE binding protein (EN-RAGE) and high mobility group box 1 (HMGB-1) are novel biomarkers in chronic kidney disease (CKD). However, their clinical significance in acute kidney injury (AKI) is unknown. The aim of this cross-sectional study was to determine whether selected biomarkers are changed in AKI patients.

**Methods:**

Serum PlGF, PAPP-A, sRAGE, EN-RAGE and HMGB-1 levels were assessed in 40 patients with AKI, 42 CKD 5 patients, 31 haemodialysis patients (HD) and 39 age-matched healthy controls.

**Results:**

PAPP-A was elevated in AKI (20.6 ± 16.9 mIU/L) compared with controls (9.1 ± 2.3 mIU/L, p < 0.001). PlGF was not increased in AKI (11.7 ± 7.4 pg/mL) versus controls (8.5 ± 2.4 pg/mL, n.s.), as well as sRAGE was not elevated in AKI (2400 ± 1400 pg/mL) compared with controls (1760 ± 730 pg/mL, n.s), but was lower compared with CKD 5 (3200 ± 1500 pg/mL, p < 0.05); EN-RAGE was elevated in AKI 480 ± 450 ng/mL in comparison with controls (60 ± 62 ng/mL), CKD 5 (190 ± 120 ng/mL), and HD (120 ± 100 ng/mL), all p < 0.001. Similarly, HMGB-1 was increased in AKI (5.8 ± 7.5 ng/mL) versus controls (1.7 ± 1.4 ng/mL), CKD 5 (3.2 ± 3.1 ng/mL) and HD (2.5 ±2.1 ng/mL), all p < 0.001.

In AKI group, in multivariate regression analysis: PAPP–A levels were associated with transferrin (p <0.001), negatively with albumin (p < 0.01) and prealbumin (p < 0.05); PlGF levels were associated with C - reactive protein (p < 0.001). EN-RAGE levels were associated with ferritin (p < 0.01) and orosomucoid (p = 0.02), and HMGB-1 levels with leukocyte count (p < 0.01) and negatively with proteinuria (p = 0.02).

**Conclusions:**

In AKI patients, PAPP-A, EN-RAGE and HMGB1 are elevated, but sRAGE and PlGF are not increased. Whereas PAPP-A correlates with markers of nutrition; PlGF, EN-RAGE and HMGB-1 are related to inflammatory parameters.

## Background

Acute kidney injury (AKI) is associated with substantial morbidity, mortality and health resource utilization [1,2]. AKI is also increasingly recognized as a prelude to chronic kidney disease (CKD) [3]. Thus, detection of patients at particular risk for death, prolonged kidney failure and associated morbidity after AKI in the setting of renal replacement therapy (RRT) remains an area of utmost interest. In addition, early identification of those likely to progress to CKD and to end stage renal disease (ESRD) and its associated cardiovascular disease (CVD) morbidity and mortality has become increasingly important. Therefore, novel validated biomarkers are required for AKI, CKD progression and associated CVD risk.

Placental growth factor (PlGF), which is a member of the vascular endothelial growth factor (VEGF), stimulates angiogenesis and growth of collateral vessels in ischemic tissues via VEGF receptor-1 (Flt1) [4,5]. PlGF is upregulated in atheromatic lesions, and antiFlt1 suppresses atherosclerotic process and plaque vulnerability [5]. Recent studies have reported that elevated levels of circulating PlGF might be associated with worsening atherosclerosis in patients with decreased renal function [6,7]. These findings have suggested that PlGF might act as an inflammatory instigator of atherosclerotic process in patients with renal impairment.

Pregnancy associated plasma protein-A (PAPP-A) is a high-molecular-weight zinc-binding metalloproteinase belonging to metzincin superfamily of metalloproteinases and was originally identified in the plasma of pregnant women [8,9]. PAPP-A was found to be abundantly expressed in eroded and ruptured vascular plaques, but is only minimally expressed in stable plaques [10]. High serum levels have been observed in patients with acute coronary syndromes [10]. PAPP-A levels are elevated in chronic haemodialysis (HD) patients and have been identified as an independent mortality predictor in long-term hemodialysis patients [11].

The receptor for advanced glycation end products (RAGE) is a member of immunoglobulin superfamily and is implicated in the pathogenesis of many diseases including vascular disease, diabetic complications or inflammatory diseases [12,13]. Advanced glycation end products and other RAGE ligands accumulate in renal failure [14]. These compounds are currently considered as likely players in atherosclerosis in patients with chronic kidney disease [15]. RAGE exists in several variants. A C-truncated variant, also known as soluble RAGE (sRAGE), is a naturally inhibitor of the ligand –RAGE interaction [16]. Serum sRAGE levels increase in patients with decreased renal function [14,16], and an inverse link between sRAGE and plaque burden in CKD have been reported [17] implicating the RAGE pathway in vascular damage in patients with decreased renal function.

The extracellular newly identified RAGE binding protein (EN-RAGE), also known as calcium binding protein S100A12, is a ligand for RAGE that is expressed on macrophages, lymphocytes and the endothelium. Binding of S100A12 to RAGE activates the proinflammatory response and is overexpressed at sites of local inflammation [18]. In patients with renal disease a relation of EN-RAGE levels to markers of inflammation was found [19]. In addition, it was suggested that elevated EN-RAGE and sRAGE levels have opposite associations with inflammation in prevalent HD patients [20].

High mobility group box-1 (HMGB-1) is a 30-kDa nuclear and cytosolic ubiquitous protein, a DNA – binding protein, known as a transcription and growth factor [21]. It has been implicated as a putative danger signal involved in the pathogenesis of a variety of inflammatory conditions [22]. HMGB-1 has been reported to trigger cellular signaling through toll-like receptor (TLR) 2, TLR4, and TLR9 and receptor for advanced glycation end products, leading to the recruitment of inflammatory cells and the release of proinflammatory cytokines and chemokines that cause organ damage [13,23,24]. Extracellular HMGB-1 is also involved in the progression of several inflammatory diseases, including septic shock [25], as well as chronic inflammatory diseases such as rheumatoid arthritis [26] and atherosclerosis [27]. More recent study in animal models demonstrated that HMGB-1 is an early mediator of kidney ischemia reperfusion injury [28]. Moreover, the only study in CKD patients has shown that HMGB-1 correlates with renal function as well as markers of inflammation and malnutrition in CKD patients [29].

In study presented here, we tested the hypothesis that the circulating PlGF, PAPP-A, sRAGE, EN-RAGE and HMGB-1 in patients with AKI are altered and might serve as biomarkers in this setting. We also examined the correlates of the studied biomarkers specifically their possible relationship to inflammation, nutrition and other parameters, whose associations are biologically plausible in AKI patients.

## Methods

### Subjects

This cross-sectional, single-centre study at the Department of Nephrology, First Faculty of Medicine, Charles University in Prague and General University Hospital in Prague, Prague, Czech Republic enrolled forty AKI patients at the inception of renal replacement therapy (RRT). Forty two patients with CKD 5 at the onset of RRT, thirty one long-term HD and thirty nine age-matched healthy control subjects served for comparison.

Written informed consent and laboratory samples were obtained from all subjects according to ethical guidelines. The study was approved by the local Institutional Ethical Committee.

Demographic and biochemical characteristics of the studied groups are presented in Table 1.

**Table 1:** Clinical and laboratory data of control subjects, AKI, CKD 5 and HD patients

**Variable**	**AKI**	**CKD5**	^**a**^**HD**	**Controls**	**pANOVA**
Number of patients (M/F)	22/18	24/18	15/16	14/25	
Age, years	58 ± 17	59 ± 13	59 ± 16	57 ± 10	0.87
BMI, kg/m^2^	28.3 ± 6.7	28.6 ± 6.9	24.3 ± 4.1	25.2 ± 3.4	<0.001
PlGF, pg/mL	11.7 ± 7.4	12.3 ± 12.4	11.5 ± 3.8	8.5 ± 2.4	0.02
PAPP-A, mIU/L	20.0 ± 16.9	20.2 ± 28.1	20.8 ± 10.1	9.1 ± 2.3	<0.006
sRAGE, pg/mL	2400 ± 1400	3200 ± 1500	2700 ± 1200	1760 ± 730	<0.0001
EN-RAGE, ng/mL	480 ± 450	190 ± 120	120 ± 100	60 ± 62	<0.0001
HMGB-1, ng/mL	5.8 ± 7.5	3.2 ± 3.1	2.5 ± 2.1	^b^1.7 ± 1.4	<0.0001
BUN, mmol/L	29 ± 13	27.1 ± 7.8	26.4 ± 7.5	4.9 ± 1.2	<0.0001
Creatinine, μmol/L	593 ±272	520 ± 140	800 ± 210	86 ± 12	<0.0001
Albumin, g/L	30.1 ± 7.0	35.5 ± 7.1	40.7 ± 3.3	43.6 ± 2.4	0.0001
Prealbumin, g/L	0.2 ± 0.1	0.26 ± 0.11	0.32 ± 0.7	0.26 ± 0.02	<0.0001
CRP, mg/L	60 ± 70	19 ± 22	12 ± 16	3.2 ± 2.1	<0.0001
Fibrinogen, g/L	5.2 ± 1.9	5.2 ± 1.4	4.8 ± 1.4	3.35 ± 057	0.002
Orosomucoid, g /L	1.6 ± 0.67	1.38 ± 0.47	1.7 ± 0.38	0.78 ± 0.19	<0.001
Hemoglobin, g/L	101 ± 22	102 ± 19	108 ± 10	141 ± 10	0.001
Leukocytes x10^9^	10.7 ± 5.3	8.5 ± 3.5	7.5 ± 2.5	6.3 ± 1.8	<0.001
Proteinuria g/ 24 hours	2.5 ± 3.8	2.8 ± 3.4	–	–	
Residual diuresis, L/ 24 hours	1.9 ± 1.3	1.8 ± 1.0	0.67 ± 0.70	–	<0.0001
GFR, mL/s/1.73 m^2^	0.18 ± 0.18	0.18 ± 0.09	0.11 ± 0.05	1.26 ± 3.0	<0.0001

Data are expressed as mean ± SD and analysed using ANOVA.

^a^Some results of HD patients were presented previously [7,19].

^b^Measured in 27 control subjects.

*Abbreviations*: *AKI* acute kidney injury, *CKD5* chronic kidney disease stage 5, *BMI* body mass index, *BUN* blood urea nitrogen, *CRP* C-reactive protein, *F* female, *M* male, *sRAGE* soluble receptor for advanced glycation end products, *EN-RAGE* extracellular newly identified receptor for advanced glycation end products binding protein, *HD* haemodialysis, *HMGB*-1 high mobility group box protein-1, *PAPP-A* pregnancy-associated plasma protein-A, *SD* standard deviation, *GFR* glomerular filtration rate.

AKI was determined using the RIFLE (Risk, Injury, Failure, Loss, and End stage kidney) staging criteria for changes in the serum creatinine within one week [30]. The enrolment was performed by attending nephrologists prior to RRT initiation. Further, blood tests and physiological parameters were obtained for each patient at the time of admission to the department after inclusion but before initiation of RRT. The aetiologies of AKI were ischemia (39.8%), nephrotoxicity (22%), and multifaceted factors (38.2%). All enrolled patients with AKI were hemodynamically stable. The patients on mechanical ventilation were not included. We included AKI patients without sepsis. Most patients received medication used in acute kidney injury including vasoactive therapy, fluid supplementation before RRT, anticoagulation, antihypertensive treatment. Eligible patients received empirical antibiotic regimens. Antibiotics were generally dosed as recommended in the corresponding package inserts. The dose of antibiotics was adjusted according to patients’ conditions and creatinine clearance.

Forty patients with CKD stage 5 with glomerular filtration rate (eGFR < 15 ml/min/1.73 m^2^) at the onset of RRT were included. The aetiology of CKD were vasculits (11%), chronic glomerulonephritis (23%) hypertension (19%) and diabetes (12%). The CKD patients were in stable clinical status, without signs of overt inflammation. Most patients received medications commonly used in patients with CKD, such as diuretics; antiplatelet drugs; calcium and vitamin D supplements; statins; and antihypertensive drugs.

Thirty one patients on maintenance haemodialysis, who had been treated at least three months, were included. Underlying renal diseases were diabetic nephropathy (12%), hypertensive nephrosclerosis (15%), polycystic disease (22%), interstitial nephritis (16%) and unknown aetiology (22%). All HD patients were receiving conventional 4-hour dialysis treatment 3 times a week with standard bicarbonate dialysis solution with heparin as anticoagulant. The average dose of dialysis (Kt/V) was 1.46 ± 0.2. The majority of patients were treated with antihypertensive medication and 45% were also treated with statins for dyslipidemia. The HD patients were in stable clinical status, without signs of overt inflammation. The detailed patients’ characteristics were published previously [7,19].

The control group consisted of thirty nine age matched healthy subjects. They were not administered any special alimentary supplements at the time of the study.

### Blood samples

In AKI and CKD 5 groups, blood was collected prior to the first dialysis session and prior to heparin administration. In HD patients, blood was collected via puncture of the arteriovenous fistula before starting the dialysis session and prior to heparin administration. In other subjects, blood was collected after overnight fasting via puncture of the cubital vein, simultaneously with blood collection for routine control examinations.

Blood count and routine biochemical parameters were determined in fresh samples. For special biochemical analyses, blood was centrifuged for 10 min at 1,450 g, and serum was frozen at −80°C until analysis. All sera were analyzed within one year.

### Laboratory parameters

PlGF was measured by means of sandwich ELISA (enzyme-linked immunosorbent assay) using standard kits (R&D Systems, Inc., Minneapolis, MN, USA) according to the manufacturer’s protocol. Results are given in picograms per milliliter (pg/mL).

PAPP-A was assessed immunochemically with TRACE (Time Resolved Amplified Cryptate Emission) by the KRYPTOR analyzer (Thermo Fischer, Henningsdorf, Germany). The results are expressed in mIU/L.

sRAGE was measured using a commercially available sandwich ELISA kit according to the instructions of the manufacturer (Quantikine; R&D Systems, Inc., Minneapolis, MN, USA). Results are given in picograms per milliliter (pg/mL).

EN-RAGE was measured by means of a sandwich ELISA using standard kits (Circulex™, CycLex Co. Ltd., Nagano, Japan) according to the manufacturer’s protocol. Results are given in nanograms per milliliter (ng/mL).

HMGB-1 was measured using a commercially available sandwich ELISA kit according to the instructions of the manufacturer (IBL International GmbH, Hamburg, Germany). Results are given in picograms per milliliter (pg/mL).

C-reactive protein (CRP) and prealbumin were determined turbidimetrically, orosomucoid (acidic α1-glycoprotein) and alpha-2 Macroglobulin were assessed nephelometrically and fibrinogen was measured by the trombin method. Albumin was determined by photometry with bromcresole green. Routine biochemical parameters and blood count were assessed using standard laboratory methods with automated analyzers. The eGFR was calculated using the MDRD formula [31].

### Statistical analysis

Statistical analyses were performed using Statistics Toolbox™ MATLAB® software (The MathWorks™, Inc., Natick, Massachusetts, USA). Data are presented as the mean ± SD for continuous variables and percentages for categorical variables. Univariate comparisons of continuous variables between control subjects and renal disease patients were conducted with unpaired sample t-tests; and ANOVA with post tests for normally distributed continuous variables. Mann–Whitney U test and Kruskal-Wallis ANOVA with Tukey-Kramer or Dunn's post tests for non-normal distributions was used to compare continuous variables between control subjects and renal patients. Variables with non-normal distributions were log-transformed where appropriate. Association among analyzed parameters was assessed using Spearman’s or Pearson’s correlation coefficient. Stepwise multivariate regression analysis was used to assess independent predictors of studied biomarkers. All results were considered statistically significant at p < 0.05.

## Results

**Figure 1:**
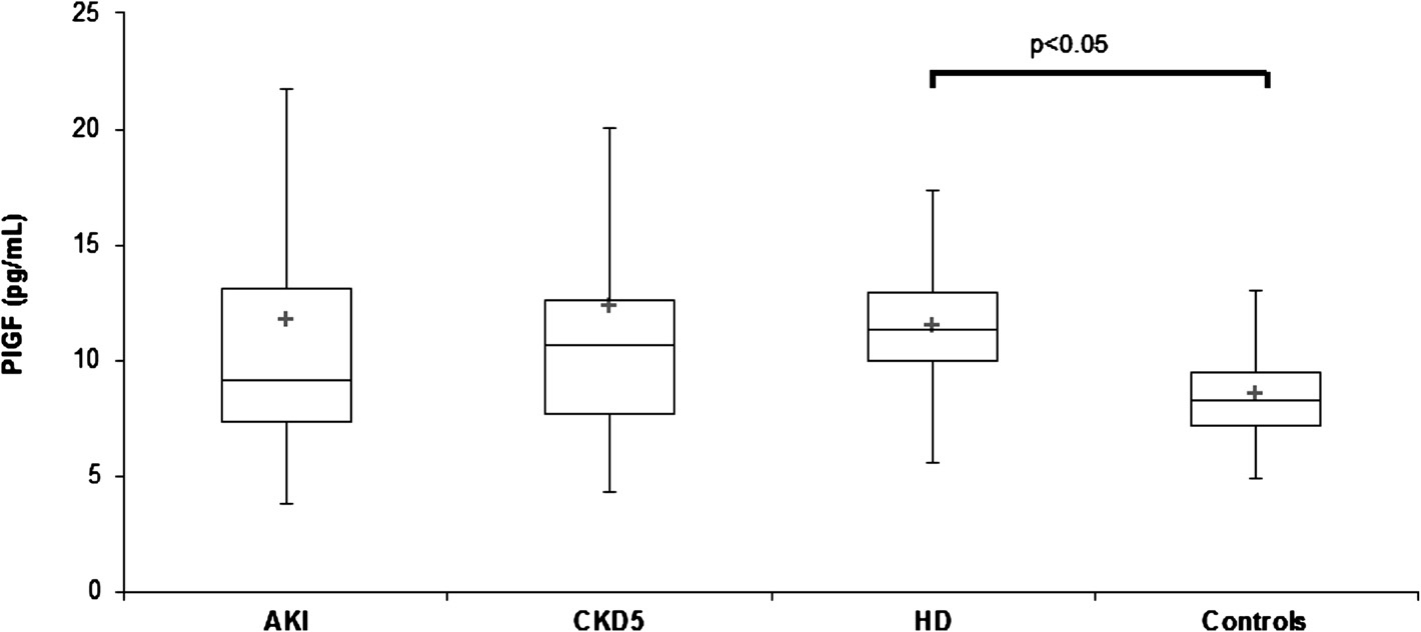
**Serum PlGF levels in AKI, CKD 5, HD and healthy controls.** Data are expressed as mean ± SD and analysed using ANOVA. Post-tests (Tukey-Kramer test or Dunn’s multiple comparison test).

**Figure 2:**
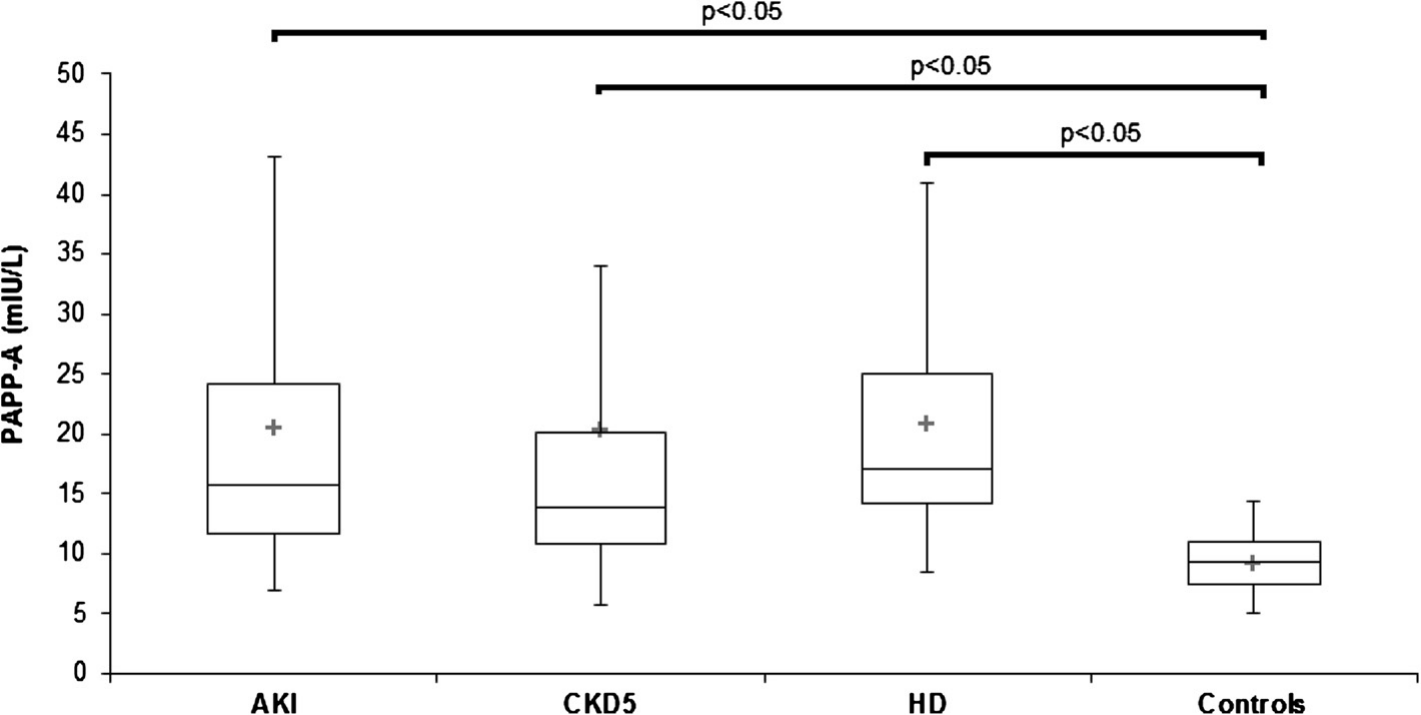
**Serum PAPP-A levels in AKI, CKD 5, HD and healthy controls.** Data are expressed as mean ± SD and analysed using ANOVA. Post-tests (Tukey-Kramer test or Dunn’s multiple comparison test).

**Figure 3:**
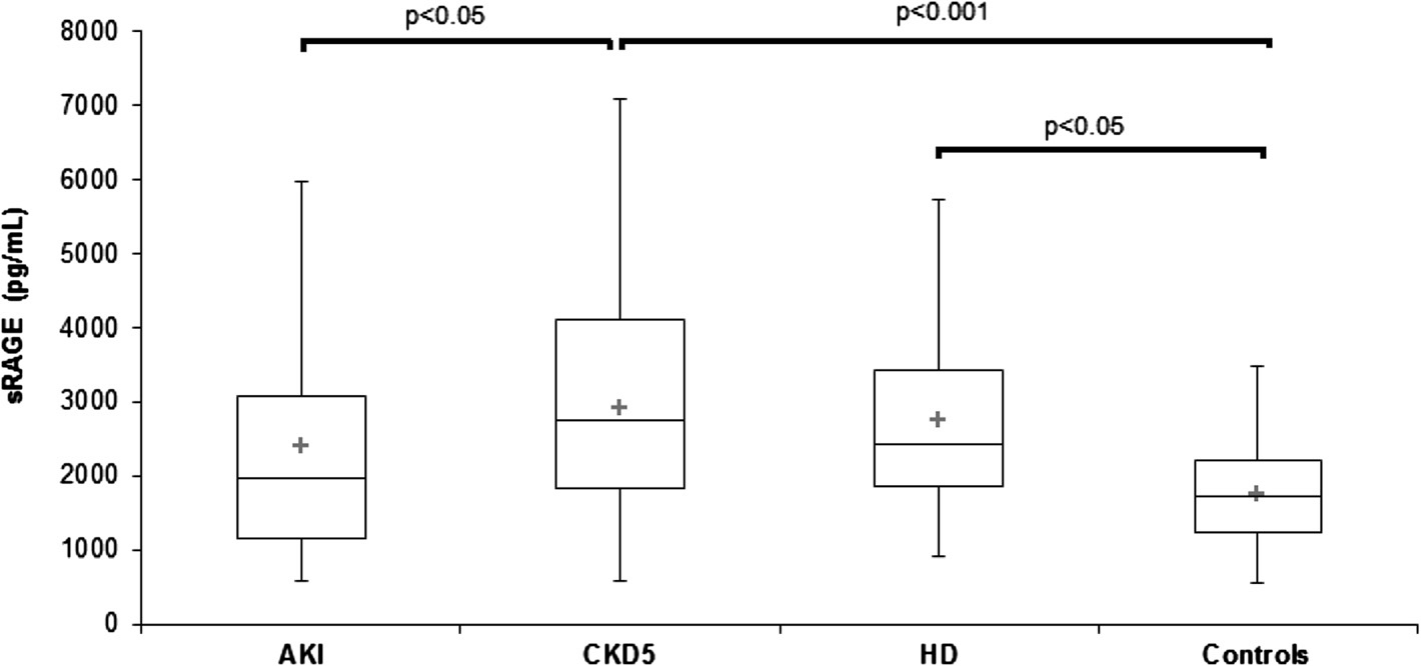
**Serum sRAGE levels in AKI, CKD 5, HD and healthy controls.** Data are expressed as mean ± SD and analysed using ANOVA. Post-tests (Tukey-Kramer test or Dunn’s multiple comparison test).

**Figure 4:**
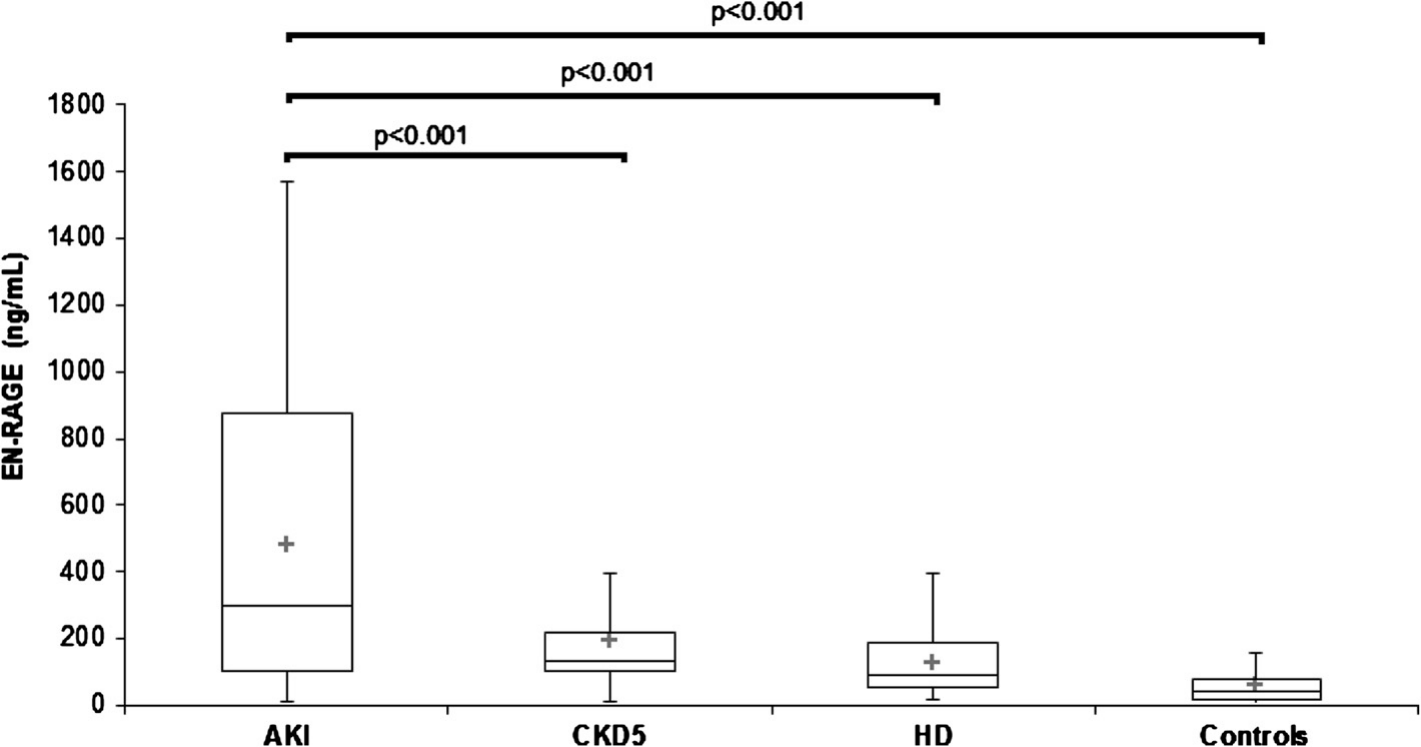
**Serum EN-RAGE levels in AKI, CKD 5, HD and healthy controls.** Data are expressed as mean ± SD and analysed using ANOVA. Post-tests (Tukey-Kramer test or Dunn’s multiple comparison test).

**Figure 5:**
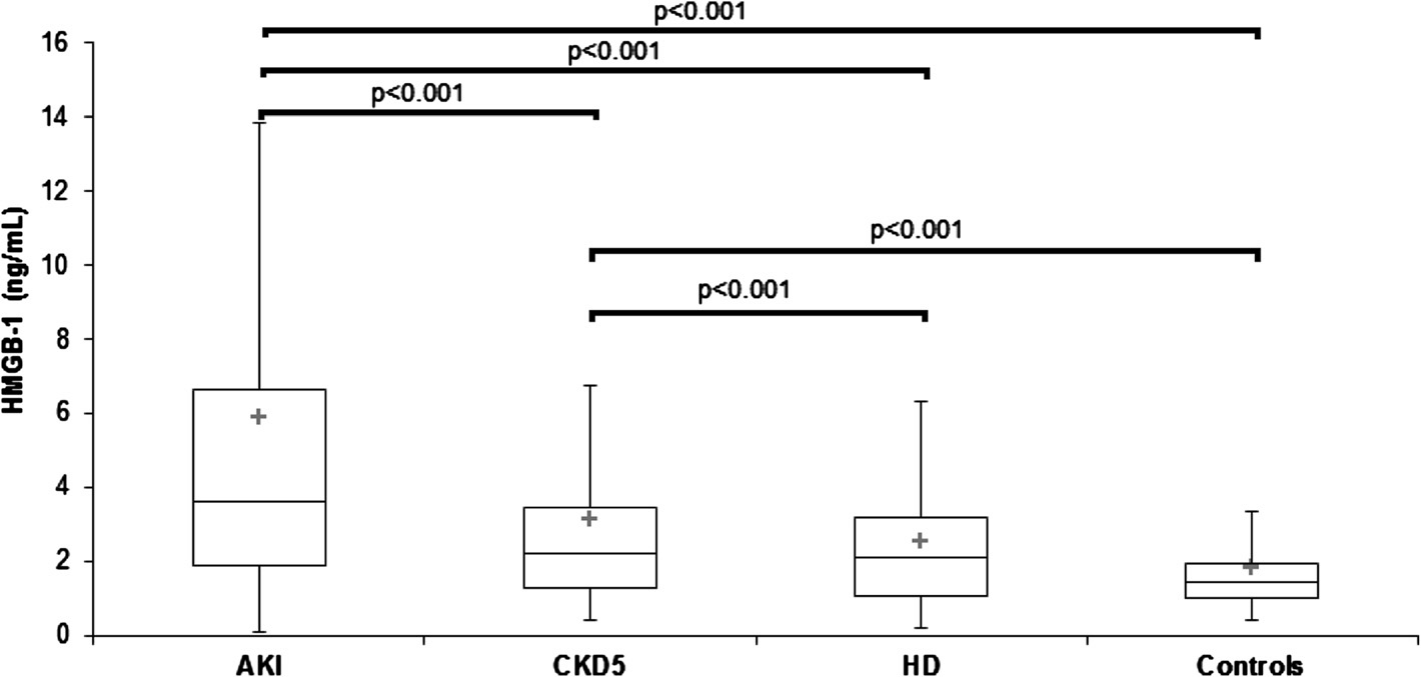
**Serum HMGB1 levels in AKI, CKD 5, HD and healthy controls.** Data are expressed as mean ± SD and analysed using ANOVA. Post-tests (Tukey-Kramer test or Dunn’s multiple comparison test).

Serum PlGF, PAPP-A, sRAGE, EN-RAGE, and HMGB-1 determined from blood obtained in AKI, CKD 5, HD and control groups are displayed at Table 1. PlGF was not increased in AKI (11.7 ± 7.4 pg/mL) compared with controls (8.5 ± 2.4 pg/mL, n.s.), but was elevated (p < 0.05) in HD (11.5 ± 3.8 pg/mL, p < 0.05) versus controls (Figure 1). PAPP-A was elevated in AKI (20.0 ± 16.9 mIU/L) CKD 5 (20.2 ± 28.1 mIU/L) and HD (20.8 ± 10.1 mIU/L) compared with controls (9.1 ± 2.3 mIU/L, p < 0.001) (Figure 2). sRAGE was not elevated in AKI (2400 ± 1400 pg/mL) compared with controls (1760 ± 730 pg/mL, n.s), but was lower compared with CKD 5 (3200 ± 1500 pg/mL, p < 0.05). sRAGE was increased in CKD 5 (3200 ± 1500 pg/mL) and HD (2700 ± 1200 pg/mL) versus controls (Figure 3). EN-RAGE was elevated in AKI (480 ± 450 ng/mL) in comparison with controls (60 ± 62 ng/mL), CKD 5 (190 ± 120 ng/mL), and HD (120 ± 100 ng/mL), all p < 0.001 (Figure 4). Similarly, HMGB-1 was increased in AKI (5.8 ± 7.5 ng/mL) versus controls (1.7 ± 1.4 ng/mL), CKD 5 (3.2 ± 3.1 ng/mL) and HD (2.5 ±2.1 ng/mL), all p < 0.001, as well as HMGB-1 was higher in CKD 5 and HD in comparison with controls (Figure 5).

The results of univariate correlations between PlGF, PAPP-A, sRAGE, EN-RAGE, HMGB-1 and other variables in AKI patients and other studied groups were shown at Table 2. In AKI group, sRAGE levels were inversely correlated with haemoglobin (r = − 0.44, p = 0.001). In multivariate regression analysis: PAPP–A levels were associated with transferrin (p <0.001), negatively with albumin (p < 0.01) and prealbumin (p < 0.05); PlGF levels were associated with C - reactive protein (p < 0.001). EN-RAGE levels were associated with ferritin (p < 0.01) and orosomucoid (p = 0.02), and HMGB-1 levels with leukocyte count (p < 0.01) and negatively with proteinuria (p = 0.02) (Table 3).

**Table 2:** Univariate correlations between biomarkers and other variables

	**AKI**	**CKD 5**	**HD**	**Controls**
PlGF	CRP,	EN-RAGE,	–	Age,
	r = 0.57, p = 0.002			
	Fibrinogen,			
	r = 0.47, p = 0.002	r = 0.34, p = 0.03		r = − 0.41, p = 0.01
	Prealbumin,			
	r = − 0.37, p = 0.02			
PAPP-A	Albumin	Fibrinogen,	Leucocyte count,	Cholesterol,
	r = − 0.42, p = 0.01	r = − 0.34, p = 0.03	r = − 0.34, p = 0.03	
	Transferin,	Serum protein,	Albumin,	
	r = 0.36, p = 0.01	r = − 0.38, p = 0.03	r = − 0.038, p = 0.03	
	Prealbumin	BUN,	CRP,	r = − 0.44, p = 0.01
	r = − 0.42, p = 0.01	r = 0.32, p = 0.03	r = 0.4, p = 0.02	
			Cholesterol,	
			r = 0.4, p = 0.02	
sRAGE	Haemoglobin,	Leucocyte count,	Ferritin,	EN-RAGE,
		r = − 0.36, p = 0.03		r = − 0.41, p = 0.01
	r = − 0.44, p = 0.001	GFR,	r = 0.43, p = 0.02	GFR,
		r = − 0.32, p = 0.02		r = − 0.37, p = 0.04
EN-RAGE	CRP,	HMGB-1,	HMGB-1,	Prealbumin,
	r = 0.36, p = 0.03			
	Orosomucoid,		r = 0.63,. p = 0.001	
	r = 0.46, p = 0.003		Fibrinogen,	
	Ferritin,		r = 0.49, p = 0.01	r = 0.39, p = 0.02
	r = 0.51, p = 0.001	r = 0.38, p = 0.04	CRP,	GFR,
	Leucocyte count,	Age,	r = 0.78, p = 0.001	r = 0.33, p = 0.04
	r = 0.51, p = 0.03	r = − 0.44, p = 0.04	Orosomucoid,	sRAGE,
	GFR,		r = 0.43, p = 0.001	r = − 0.41, p = 0.01
	r = − 0.34, p = 0.04		Leukocyte count,	
	BUN,		r = − 0.56, p = 0.01	
	r = 0.33, p = 0.03			
HMGB-1	Leucocyte count,	Leucocyte count,	CRP,	–
	r = 0.42, p = 0.01			
	Proteinuria,		r = 0.45, p = 0.01	
	r = − 0.36, p = 0.02	r = 0.48, p = 0.001	Total protein,	
	Cholesterol,		r = 0.48, p = 0.01	
	r = − 0.34, p = 0.03			

Data are expressed as mean ± SD.

*Abbreviations*: *AKI* acute kidney injury, *BUN* blood urea nitrogen, *CKD5* chronic kidney disease stage 5, *CRP* C-reactive protein, *sRAGE* soluble receptor for advanced glycation end products, *EN-RAGE* extracellular newly identified receptor for advanced glycation end products binding protein, *HD* haemodialysis, *HMGB-1* high mobility group box protein-1, *PAPP-A* pregnancy-associated plasma protein-A, *SD* standard deviation, *GFR* glomerular filtration rate.

**Table 3:** Associations of PlGF, PAPP-A, EN-RAGE and HMGB-1 levels in AKI patients (multivariate regression analysis)

	**Predictor**	**B coefficient**	**SE**	**T**	**p**	**Intercept**	***R***^**2**^
PlGF	CRP	0.0018	0.00043	4.2	0.0001	0.8	0.32
PAPP-A	Albumin	−0.0153	0.0048	−3.1	0.003	1.6	0.47
	Transferrin	0.0674	0.0174	3.8	0.0004		
	Prealbumin	−0.6379	0.3005	−2.1	0.04		
EN-RAGE	Ferritin	519.26	181.72	2.8	0.006	−955.48	0.35
	Orosomucoid	722.37	318.18	2.2	0.02		
HMGB-1	Leucocyte count	1.063	0.384	2.7	0.008	−0.537	0.28
	Proteinuria/24 hours	−0.307	0.128	−2.3	0.02		

*Abbreviations*: *CRP* C-reactive protein, *sRAGE* soluble receptor for advanced glycation end products, *EN-RAGE* extracellular newly identified receptor for advanced glycation end products binding protein, *HMGB-1* high mobility group box protein-1, *PAPP-A* pregnancy-associated plasma protein-A.

To conclude the PAPP-A, EN-RAGE and HMGB-1 levels are significantly elevated, but sRAGE and PlGF levels are not increased in AKI patients. sRAGE has a reverse relation to haemoglobin. PAPP-A levels are independently associated with markers of nutrition: transferin and negatively with albumin and prealbumin. PlGF is associated with CRP. EN-RAGE is independently associated with inflammatory markers: ferritin and orosomucoid. HMGB-1 is associated with leukocyte count and negatively with proteinuria in AKI patients.

## Discussion

This is the first study where we demonstrate the circulating levels of PLGF, PAPP-A, sRAGE, EN-RAGE and HMGB-1 levels in patients with AKI requiring RRT. Significantly higher levels of PAPP-A, EN-RAGE and HMGB-1, but not increased levels of sRAGE and PlGF were observed in the serum of patients with AKI as compared with controls. Further, this study demonstrates significant independent associations of PAPP-A with markers of nutrition, and the associations of PlGF, EN-RAGE, and HMGB-1 with inflammatory parameters in these patients for the first time.

Although PlGF levels in AKI patients were not elevated, PlGF was significantly correlated with inflammatory markers CRP and fibrinogen and inversely with a negative inflammatory marker prealbumin. However, only CRP was positively associated with PlGF levels by multivariate analysis. CRP is a short pentraxin and an established biomarker of inflammation in kidney disease [32]. A recent study has suggested that the level of the ratio of CRP to prealbumin was associated with mortality of AKI patients [33]. Moreover, lower serum prealbumin levels were strongly associated with a higher risk of death independent of AKI severity [34]. On the other hand, serum fibrinogen is independently predictive of cardiovascular and all-cause mortality in end-stage kidney disease [35] and in patients with CKD [36]. In AKI serum fibrinogen levels were comparable with those found in healthy controls [37]. It is thus conceivable that PlGF is released from endothelial cells, among others, in response to inflammation in AKI.

PAPP-A levels were increased in AKI patients in comparison with healthy controls, but were comparable to those found in CKD 5 and HD patients. In line with previous report, PAPP-A is elevated in HD patients [38] and is a prognostic marker in dialysis patients [11]. The PAPP-A levels were also significantly decreased in dialysis patients after successful kidney transplantation, but remained higher than in control group [39]. The mechanisms of PAPP-A increase most probably include the increased synthesis, but also the decreased clearance of PAPP-A in patients with decreased renal function, including the patients with AKI. In this study, PAPP-A levels were independently associated with markers of nutrition: transferin and negatively with albumin and prealbumin. These results permit the conclusion that PAPP-A levels are elevated in patients with AKI and related to markers of nutrition, but are not related to inflammatory markers, as in HD patients in this and previous studies [40].

We provide here evidence that sRAGE levels are increased but not significantly in the setting of AKI. An explanation for the comparable sRAGE levels in AKI might be an enhanced consumption of this molecule. sRAGE acts as an anti-inflammatory “decoy” by binding and preventing their interaction with cell surface RAGE, suppresses the RAGE mediated inflammatory response [16]. The ligands EN-RAGE and HMGB-1 binding to sRAGE might influence the levels of sRAGE and increase the propensity towards inflammation. RAGE ligands therefore have better binding across to cell membrane receptor, the binding of which activates the inflammatory pathways. Interestingly, in a recent study in septic AKI patients sRAGE levels were elevated [41]. In CKD and HD patients serum sRAGE levels were also increased in this and the previous study and was inversely related to inflammation [42]. The correlation revealed in our AKI patients between serum sRAGE levels and declining haemoglobin suggest that reduced tissue oxygenation associated with anaemia may contribute to the formation of AGEs and activation of RAGE with possible toxic effect of them on haematopoiesis, while sRAGE might inhibit their pathological effect. We cannot also exclude the effect of amelioration of endothelial and inflammatory injuries on the serum sRAGE activity in AKI. sRAGE levels in AKI, similarly as in CKD and HD, could be an indicator of enhanced RAGE expression as counter-regulatory system against endothelial damage i.e. inflammation and oxidative stress. Given the importance of anaemia in decreased renal function, the association between sRAGE and anaemia in AKI patients deserves further studies.

In the present study, EN-RAGE levels were significantly increased in AKI patients, but not in CKD5 and HD patients. These results are in line with our previous study where the serum concentrations of CKD patients and HD were not elevated in comparison with healthy controls [19]. Similarly as in CKD, HD and peritoneal dialysis patients [19,43], also in AKI patients a relation of serum EN-RAGE levels to markers of inflammation was found. Specifically, EN-RAGE concentrations were independently associated with orosomucoid and ferrtitin.

Plasma EN-RAGE triggers the RAGE pathway as proinflammatory ligand activating key inflammatory signals such as NF-κB and MAP kinase and stimulates cell adhesion molecules. Circulating EN-RAGE is associated with CVD events and CVD-related mortality in HD patients, which partly explained by its link to inflammation [44], and is related to mortality of HD patients due to infection [45]. Orosomucoid, being an acute phase protein, contributes to immune response in inflammatory states modulating chemotaxis of neutrophils, superoxide generation and aggregation [46]. On the other hand, a recent study in a murine model of acute renal failure has shown that orosomucoid partially restored activity of clotting and complement systems in acute renal failure [47]. This effect may be due to accumulation of orosomucoid in renal tissue and its protective action in situ. Taken together, higher serum EN-RAGE levels and relation to inflammatory markers in this study may be associated with amplified inflammatory response and vascular damage in AKI patients.

In the present study all AKI patients in our study had elevated circulating HMGB-1 levels as compared with controls. We could also show that HMGB-1 levels were independently associated with leukocyte count and negatively with proteinuria in AKI setting. Although, we could not exclude patients with high CRP levels in AKI patients, in multivariate analysis no relationship to CRP levels were found. HMGB-1 is one of the high-affinity ligands for RAGE/sRAGE, a potent cytokine playing an important role in the pathogenesis of inflammation. Previous studies have shown that HMGB-1 differs from early innate proinflammatory cytokines, such as TNF and IL-1, in endotoxaemia and sepsis models [25,48]. HMGB-1 release occurs in response to a number of alarm signals including endotoxin, interferons, TNFs and largely is a consequence of NF-κB activation and HMGB-1 acetylation at its nuclear localization site [49,50]. This induces vesicular sequestration and leads to extracellular HMGB-1 release [51,52]. In addition, passive diffusion from necrotic cells might occur [51,53]. Another interesting finding is the negative association of HMGB-1 and proteinuria in AKI setting, supporting the concept that HMGB-1 could be a marker of renal injury in patients with AKI. Whether high HMGB-1 levels in AKI are the consequences of the disease or a potential contributing factor to the disease needs to be elucidated.

The most frequent cause of AKI in the Intensive Care Units is sepsis [54]. Endothelial activation defined as upregulation of adhesion molecules by proinflammatory cytokines, may be central to the development of sepsis induced AKI.

In this study the CKD and HD patients with overt inflammation were excluded. We endeavored to include a comparative cohort of AKI patients specifically without sepsis. Although, we have not included the patients with sepsis in this study, the association of studied biomarkers with inflammatory markers support the notion that also in sepsis induced AKI the levels of studied biomarkers might be changed. Indeed, pretransplant inflammation including the elevation of PAPP-A in transplant recipients might play an important role in the pathogenesis of ischemic AKI and could be a risk factor for the development of delayed graft function [55]. Serum PAPP-A levels frequently increases in patients with severe sepsis and appears to be associated with sepsis related myocardial dysfunction [56]. PlGF levels are elevated in preclinical models of sepsis [57]. PlGF protects liver endothelial cells against septic injury, explaining why sepsis morbidity is increased following genetic or pharmacological PlGF blockade [57,58]. sRAGE levels were elevated during acute lung injury, regardless of the presence or absence of severe sepsis [59]. Also in another study in septic patients an elevation of sRAGE levels were shown [60]. Non-survivors had higher plasma sRAGE concentrations than survivors. In addition, recently also in septic AKI patients sRAGE levels were elevated [41]. In contrast, in a recent study the sRAGE levels were not changed in severe sepsis, while the EN-RAGE concentrations were significantly increased in patients with severe sepsis stratified to the three most common infectious sources (lungs, abdomen, and urinary tract) [61]. In addition, HMGB-1 has been identified as late cytokine mediator of endotoxaemia and sepsis [52,62,63]. HMGB-1 was persistently elevated in patients with severe sepsis and severe shock [64]. Taken together, PlGF, PAPP-A, sRAGE, EN-RAGE and HMGB-1 might play a role also in sepsis induced AKI. Further studies are warranted to test the clinical utility of these biomarkers in managing patients with sepsis and AKI and to better understand their relationship with kidney morphology during acute kidney injury.

There are several limitations in this study, including small sample size of adult patients with severe AKI (RIFLE category failure). Nevertheless, this is the first study to report an association of studied biomarkers and relevant parameters in AKI patients. Second, the studied population was composed by heterogeneous AKI patients treated at single centre of faculty hospital. Third, we did not compare studied biomarkers with established one such as neutrophil gelatinase associated lipocalin. Finally, we did not perform a kinetic study on novel biomarkers including more frequent sampling.

## Conclusions

The study presented here provides first insight into levels of circulating PlGF, PAPP-A, sRAGE, EN-RAGE and HMGB-1 in patients with AKI. The PAPP-A, EN-RAGE and HMGB1 levels are significantly elevated, but sRAGE and PlGF levels are not increased in AKI patients. Whereas PlGF, EN-RAGE, and HMGB-1 levels are significantly related to inflammatory markers, PAPP-A levels are associated with markers of nutrition in AKI setting. Larger, prospective clinical studies are needed to confirm the results of our single centre study.

## Competing interests

The authors declare that they have no competing interests.

## Authors’ contributions

OZ participated in sample collection, clinical data collection, laboratory processing and drafted the manuscript. VK participated in the design of the study and performed the statistical analysis. JV was inestimable in sample collection and clinical data collection. TZ and VT provided expert opinion, took part in data interpretation and manuscript preparation. MK conceived the study, and participated in biochemical analysis of the samples, participated in design and coordination and helped to draft the manuscript. All authors read and approved the final manuscript.

## Pre-publication history

The pre-publication history for this paper can be accessed here:

http://www.biomedcentral.com/1471-2369/14/245/prepub
